# Rare KDM4C variants and reduced expression underlie epigenetic dysregulation in rheumatoid arthritis

**DOI:** 10.1007/s12026-026-09751-9

**Published:** 2026-02-13

**Authors:** Deniz Aslar Oner, Hasan Toktas, Umit Dundar, Sevda Adar, Nuran Eyvaz

**Affiliations:** 1 Atatürk Vocational School of Health Services, Afyonkarahisar Health Sciences University, Afyonkarahisar, Turkey; 2 Faculty of Medicine, Department of Physical Therapy and Rehabilitation, Afyonkarahisar Health Sciences University, Afyonkarahisar, Turkey

**Keywords:** Mutation, missense, Gene expression, Epigenetics, Arthritis, rheumatoid, Histone demethylases, KDM4C protein, human

## Abstract

This study aims to investigate structural and expression-level alterations in the histone demethylase *KDM4C* gene in patients with rheumatoid arthritis (RA) and elucidate its role in the disease’s epigenetic basis. *KDM4C* sequencing was performed in RA patients, and identified variants were mapped to functional domains, including the JmjN and PHD-type2 regions. *KDM4C* expression was assessed by quantitative PCR in RA and control groups. Molecular findings were analyzed alongside patients’ clinical characteristics and treatment histories. A rare missense mutation (c.79 C > T, R27W) located in the JmjN domain was detected in a single patient with RA. In addition, a synonymous variant was identified in the PHD-type2 domain. The R27W variant is predicted by in silico analyses to impair protein homodimerization, while the synonymous change has been hypothesized to influence translational efficiency. Neither variant has previously been linked to RA. *KDM4C* mRNA expression was significantly reduced in patients with RA. This reduction was particularly evident in individuals with high CRP/ESR levels, positive RF and anti-CCP status, and treatment resistance. Taken together, the molecular and clinical findings suggest a potential functional alteration of *KDM4C* activity. Specifically, *KDM4C* may have a reduced capacity to remove repressive methylation marks on histone H3, particularly H3K9me3. These findings suggest that *KDM4C* dysregulation may promote a closed chromatin conformation in immune cells. This state may lead to silencing of genes involved in immune regulation and increased expression of inflammatory genes, thereby contributing to the pathogenesis of RA. *KDM4C* appears to be an important epigenetic regulator in RA pathogenesis. The coexistence of structural variants and decreased expression underscores its potential as a diagnostic biomarker and therapeutic target. To our knowledge, this is the first study providing integrated clinical and genetic evidence linking *KDM4C* dysregulation to RA.

## Introduction

Rheumatoid arthritis (RA) is a chronic inflammatory disorder that arises from complex interactions between genetic susceptibility and environmental factors. There is familial transmission in this disease, and the heritability of RA is estimated to be approximately 60–65% [31]. Advances in genome-wide association studies (GWAS) have enabled the identification and simultaneous evaluation of multiple RA susceptibility genes, including HLA-DRB1, PTPN22, CTLA4, and PADI4. These findings demonstrate that genetic factors play a central role in RA risk and disease development [9, 20].

In addition to genetic predisposition, environmental factors such as smoking, alcohol consumption, silica, asbestos, textile dust, periodontitis caused by *Porphyromonas gingivalis*, and vitamin D deficiency have also been shown to be associated with the pathogenesis of RA [16, 21, 32].

Increasing evidence indicates that epigenetic mechanisms play a substantial role in the etiology of RA. These mechanisms are involved in genome rearrangement, control of gametogenesis and early embryogenesis, and play an important role in cell differentiation [33].

Epigenetic dysregulation is a recognized contributor to a wide range of multifactorial conditions, including congenital anomalies, hereditary disorders, and cancer. In RA, multiple epigenetic mechanisms contribute to disease pathogenesis, including DNA methylation, histone modifications, epigenetic writers and erasers, and non-coding RNAs (ncRNAs). These mechanisms regulate inflammatory signaling and matrix-degrading pathways [34, 36]. Unlike DNA mutations, these alterations do not change gene structure. However, they can profoundly affect transcriptional activity [17, 41].

Histone demethylation is one of the major epigenetic modifications involved in epigenetic regulation. This process is mediated by various histone demethylases and methyltransferases. *KDM4C* has recently been identified as a member of the histone demethylase family. *KDM4C* (Histone lysine demethylase 4 C), also known as *JMJD2* (Jumonji Domain-Containing 2 C) and *GASC1* (Gene Amplified In Squamous Cell Carcinoma 1 Protein), is a member of the Jumonji domain 2 family [26].

The primary function of *KDM4C* is to remove methyl groups from trimethylated lysine 9 on the N-terminal tail of histone H3 (H3K9me3). This activity leads to transcriptional activation of target genes. Through these chromatin modifications, *KDM4C* influences heterochromatin formation, genomic imprinting, and X-chromosome inactivation. It also regulates gene transcription by altering chromatin structure [13, 19, 26, 30]. Identification of molecular biomarkers involved in RA pathogenesis is important for early diagnosis and facilitates the development of targeted therapeutic approaches. Genome-wide and locus-focused studies of fibroblast-like synoviocytes (FLS) and immune cells have demonstrated widespread alterations in DNA methylation and histone modifications, including both repressive and activating H3 marks. These epigenetic changes shape pathogenic transcriptional programs in the synovium and peripheral blood [3, 22, 45].

In particular, methylation of histone H3 at lysine 9 (H3K9) is a conserved epigenetic mark that is tightly associated with heterochromatin formation and transcriptional silencing. Dysregulated H3K9 methylation has been linked to aberrant inflammatory gene expression in chronic inflammatory diseases [11, 38]. These observations place enzymes that write or erase H3K9 methylation at the center of the epigenetic landscape of RA.

*KDM4C* is a member of the Jumonji domain-containing KDM4 family. It specifically demethylates di- and trimethylated H3K9 (H3K9me2/me3), thereby reversing a key repressive histone mark [26]. Beyond its established roles in cancer and development, accumulating evidence indicates that *KDM4C* also participates directly in immune regulation. In B cells, T follicular helper cell-derived signals induce *KDM4A* and *KDM4C* expression, which is accompanied by a global reduction in H3K9me2/3 levels. Depletion of KDM4C potentiates B-cell activation and proliferation. In addition, *KDM4A* and *KDM4C* form a complex with NF-κB p65 and WDR5 to regulate cell-cycle–associated genes. This epigenetic cascade is dysregulated in B cells from patients with systemic lupus erythematosus. These findings highlight a functional role for *KDM4C* in adaptive immune responses and autoimmunity [14]. In CD4⁺ T lymphocytes from patients with latent autoimmune diabetes in adults, reduced global H3K9 methylation has been associated with downregulation of the H3K9 methyltransferase SUV39H2 and upregulation of *KDM4C*. These findings implicate an imbalance between H3K9 writers and erasers, including *KDM4C*, in autoimmune pathogenesis [29]. Moreover, cross-phenotype Immunochip analyses have identified *KDM4C* as a shared genetic susceptibility locus in systemic vasculitides. These data provide genetic evidence that *KDM4C* contributes to the risk of immune-mediated inflammatory diseases [35]. In addition to these immune-related findings, *KDM4C* has been shown to be required for appropriate stress and injury responses in vivo. Loss of *KDM4C* perturbs autophagy, mitochondrial homeostasis, and inflammatory signaling in renal tissues [37].

Taken together, available evidence supports a role for *KDM4C* in linking H3K9 methylation dynamics with immune-cell activation, providing a biologically plausible rationale for its investigation in rheumatoid arthritis. *KDM4C* has not been identified as a top-ranked locus in rheumatoid arthritis genome-wide association studies, it has well-established roles in H3K9 demethylation and immune-cell activation. These functions provide a strong biological rationale for investigating *KDM4C* in RA. We therefore hypothesized that rare coding variants and reduced expression of KDM4C may contribute to epigenetic dysregulation in RA. This hypothesis was formulated despite the absence of strong genome-wide association signals for *KDM4C* in RA. On this basis, *KDM4C* was selected as a candidate histone demethylase for detailed genetic and expression analyses in patients with rheumatoid arthritis.

Therefore, this study aimed to investigate genetic alterations and expression levels of the histone demethylase *KDM4C* in the pathogenesis of rheumatoid arthritis. In addition, the results were correlated with relevant clinical data. To our knowledge, this is the first study to comprehensively investigate *KDM4C* gene mutations and expression profiles in patients with rheumatoid arthritis.

## Materials and methods

### Study design and participants

Patients diagnosed with RA according to the American College of Rheumatology (ACR) 2010 criteria were included in the study. Demographic and clinical information of the patients were collected during the examination. The study was approved by the Clinical Research Ethics Committee of Afyonkarahisar Health Sciences University (approval number: 2011-KAEK-2). All participants provided written informed consent. Peripheral blood samples were obtained from thirty volunteers aged 18–65 years who were diagnosed with rheumatoid arthritis according to ACR criteria. Individuals with known systemic, inflammatory, infectious, or malignant diseases other than rheumatoid arthritis were excluded. Peripheral blood samples from ten healthy volunteers without any autoimmune disease were used as the control group. RA disease activity was assessed using the DAS28 score. This assessment incorporated tender and swollen joint counts, RF, ESR, CRP, ANA, and anti-CCP antibody levels.

### Mutation screening

Genomic DNA was extracted from 2 mL of peripheral blood obtained from patients with RA and healthy controls using the Exgene Blood SV kit (GeneAll, Korea). KDM4C coding exons were PCR-amplified using specific primers (Table 1). Each 20 µL reaction mixture contained 2× Master Mix, primers (10 µM), and template DNA. PCR cycling conditions were as follows: initial denaturation at 95 °C for 5 min; 30 cycles of denaturation at 94.5 °C for 30 s, annealing at 67 °C for 30 s, and extension at 72 °C for 30 s; followed by a final extension at 72 °C for 5 min.

**Table 1:** Exon-specific primer sequences for KDM4C

Primer Name		Primer Sequence	Amplicon, bp
1-KDM4C-F	GCTGGGGGAGCTGACATACT		330
1-KDM4C-R	CAATGTCCCAAATCGTGAAA		
2-KDM4C-F	TTTTGGTGGATAGTGGTTTGA		472
2-KDM4C-R	TGGGGTTTATGGTTTCCAAG		
3-KDM4C-F	TGTTCTTCTCTGTTTTAACCTTCC		295
3-KDM4C-R	GCACATTATGTCTGGGAGGTAG		
hs-KDM4C-F (mRNA)	CCAGCAAGATCTCCGATGAC		156
hs-KDM4C-R (mRNA)	AGCTGCGAAGTTAGGGGACT		
hs-GAPDH- F	ACCCACTCCTCCACCTTTGAC		100
hs-GAPDH- R	TGTTGCTGTAGCCAAATTCGTT		

Primer sequences used for PCR amplification of *KDM4C* genomic regions and mRNA transcripts. *KDM4C*, lysine demethylase 4 C; *GAPDH*, glyceraldehyde-3-phosphate dehydrogenase; F, forward; R, reverse; bp, base pairs; mRNA, messenger RNA.

PCR products were visualized on 2% agarose gels, purified (Expin Gel SV, GeneAll), and subjected to bidirectional Sanger sequencing on an ABI 3500 Genetic Analyzer (Applied Biosystems). Sequence chromatograms were analyzed with DNA Dragon software and aligned to the *KDM4C* reference sequence (Ensembl, GRCh38) to identify nucleotide variants [12]; [8].

### *In silico* analysis of ***KDM4C*** mutations

The functional significance of the identified *KDM4C* variants was evaluated using three independent in silico tools. MutationTaster was applied to predict pathogenicity and potential splicing effects. PolyPhen-2 was used to assess the structural and functional impact of missense substitutions based on evolutionary conservation and protein context. SNAP2, a neural network-based classifier, was employed to discriminate between functionally neutral and deleterious amino acid changes [1, 6, 43].

### Variant calling and analysis

Variants in *KDM4C* were identified by PCR-Sanger sequencing. Sequences were aligned to the Ensembl reference genome (GRCh38) and validated using DNA Dragon software. Functional annotation included population frequency data from gnomAD (release 4), ClinVar classification, and CADD (v1.7) scores. Variant interpretation was performed according to ACMG/AMP guidelines [7, 23, 39, 44]. A minor allele frequency < 0.01 and CADD PHRED > 20 were considered supportive of pathogenicity. Domain localization (JmjN, PHD-type 2) was incorporated into ACMG-based classification.

Using this integrative approach, two variants were identified (Table 2). A rare missense mutation, c.79 C > T (p.Arg27Trp), was detected in the JmjN domain and classified as Likely Pathogenic (PM1, PM2, PP3). In addition, a synonymous variant, c.2556 C > T (p.Asp852=), was identified in the PHD-type 2 domain and classified as Likely Benign (BA1, BP4). These results provide a comprehensive molecular interpretation consistent with ACMG standards.

**Table 2:** Rare and functional KDM4C variants identified in RA: population frequency, in silico prediction, and ACMG classification

HGVS (DNA/Protein)	Chromosomal Position (GRCh38)	Protein Domain	dbSNP (RS ID)	ClinVar Classification	gnomAD AF	CADD PHRED Score	ACMG/AMP Classification
KDM4C(NM_001146695.4):c.79 C > T p.(Arg27Trp)	9:6793067 (GRCh38)	JmjN domain	rs772334289	Not reported	0.00001	25.5	Likely Pathogenic
KDM4C(NM_015061.6):c.2556 C > T p.(Asp852=)	9:7103816 (GRCh38)	PHD-type 2 domain	rs3763651	Not reported	0.47	1.55	Likely Benign

The functional impact of each variant was evaluated using a combination of bioinformatic algorithms accessed via the VarSome platform. Variants classified as pathogenic are those for which the majority of computational tools converge on predictions of deleterious or damaging effects, while benign classifications are supported by consistent in silico evidence indicating a lack of functional consequence. *HGVS* Human Genome Variation Society, *GRCh38* Genome Reference Consortium Human Build 38, *dbSNP* Single Nucleotide Polymorphism Database, *AF* allele frequency, *CADD* Combined Annotation Dependent Depletion, *ACMG/AMP* American College of Medical Genetics and Genomics/Association for Molecular Pathology.

### ***KDM4C*** gene expression analysis

Total RNA was extracted from peripheral blood using the Hybrid-RT Blood RNA Kit (GeneAll, Korea). Whole blood was selected for KDM4C expression analysis for practical and clinical reasons, as it is minimally invasive and routinely obtainable during follow-up visits, enabling inclusion of well-characterized RA cohorts. The aim of this analysis was to detect whether systemic differences in KDM4C transcript levels are present between RA patients and healthy controls, rather than to define cell-type–specific regulatory mechanisms. RNA concentration and purity were assessed via NanoDrop. *KDM4C* transcript levels were quantified by RT-qPCR with GAPDH as the reference gene, using SYBR Green chemistry on a QuantStudio5 system. Relative expression was calculated by the 2^-ΔΔCt method [24].

Peripheral blood for RNA isolation was drawn at routine rheumatology follow-up visits from patients with established RA who were receiving ongoing treatment with conventional synthetic DMARDs, glucocorticoids and/or biologic agents, as detailed in the clinical characteristics and treatment section. Treatment-naïve patients at initial diagnosis were not included in this cohort.

Therefore, because whole blood is a heterogeneous tissue and no cell sorting or enrichment was performed, the observed expression differences reflect an aggregate signal across circulating blood cells and may, in part, be influenced by differences in cellular composition rather than cell type–specific regulation.

### Statistical analysis

Analyses were performed using SPSS v22.0 (IBM, Armonk, NY, USA). Data distribution was assessed by the Shapiro-Wilk test. Continuous variables were summarized as mean ± SD or median, and group comparisons were made using the Mann-Whitney U test where appropriate. Categorical variables were compared by χ² test. Associations between variants and clinical features were estimated by odds ratios (OR) with 95% confidence intervals (CI). Statistical significance was set at *p* < 0.05.

## Results

### Demographic and clinical analysis of patients with RA

A total of 30 patients with RA (23 female, 7 male), aged between 18 and 65 years (mean age: 53.7 ± 11.9 years), were included in the study. The demographic and clinical characteristics of the patients are summarized in Table 3.

**Table 3:** Demographic and clinical data of rheumatoid arthritis patients

Features (Mean ± SD or n (%))	RA Patients (*n* = 30)
Age (years)	53.7 ± 11.9
Gender (Female/Male)	23/7
CRP (mg/dL)	4.05 (0.5–30.1)
ESR (mm/h)	20 (5–53)
RF Positivity (%)	53%
Anti-CCP Positivity (%)	53%
ANA Positivity (%)	37%
Disease Duration (years)	15 (1–30)
Morning Stiffness (minutes)	1.5 (0–60)
Swollen Joint Count	0 (0–1)
Tender Joint Count	0 (0–12)
DAS28 Score	2.17 ± 0.70
Family History (RA)	6 (%20)
Diabetes	4 (%13)
Hypertension	6 (%20)
Medication Use	
— DMARD — Steroid — DMARD+ Steroid — Anti-TNF — DMARD + Anti-TNF — DMARD+Steroid+Anti-TNF	5 1 14 4 4 2

Demographic, clinical, and treatment characteristics of patients with rheumatoid arthritis. Continuous variables are presented as mean ± SD for normally distributed data and as median (min–max) for non-normally distributed data. Categorical variables are presented as n (%). Abbreviations: *CRP* C-reactive protein, *ESR* erythrocyte sedimentation rate, *RF* rheumatoid factor, *anti-CCP* anti-cyclic citrullinated peptide antibody, *ANA* antinuclear antibody, *DAS28* Disease Activity Score-28, *DMARD* disease-modifying antirheumatic drug, *TNF* tumor necrosis factor

Of the 30 patients with RA, 16 (53%) were anti-CCP positive and 14 (47%) were anti-CCP negative. The same distribution was observed for RF, with 16 positive and 14 negative patients. RF and anti-CCP positivity were significantly correlated (*p* < 0.005).

CRP and ESR levels, although frequently used as acute-phase markers, showed no significant association with DAS28 scores (*p* = 0.201 and *p* = 0.281, respectively). In contrast, both CRP and ESR correlated positively with disease duration (*p* = 0.04 and *p* = 0.01, respectively). Gender-specific differences were also observed, with men exhibiting higher CRP levels than women (*p* = 0.002).

A statistically significant positive relationship was detected between RF positivity or negativity and Anti-CCP (*p* < 0.01). Anti-CCP positivity showed no relationship with disease duration (*p* = 0.66) or morning stiffness (*p* = 0.06). Similarly, RF positivity was not associated with disease duration (*p* = 0.73), but correlated with morning stiffness (*p* < 0.01).

Anti-CCP antibody levels were significantly associated with diabetes (*p* = 0.03). No significant association was observed with family history (*p* = 0.39) or hypertension (*p* = 0.26). ANA positivity was significantly associated with family history (*p* = 0.01) and hypertension (*p* = 0.04), but not with diabetes (*p* = 0.53). Finally, disease duration correlated positively with treatment duration (DMARD, steroid, anti-TNF; *p* = 0.012).

### Mutation analysis of the ***KDM4C*** gene

Two distinct variants were identified within different functional domains of the *KDM4C* gene, each with unique potential implications for RA pathogenesis.

### R27W missense variant in the JmjN domain

**Fig. 1:**
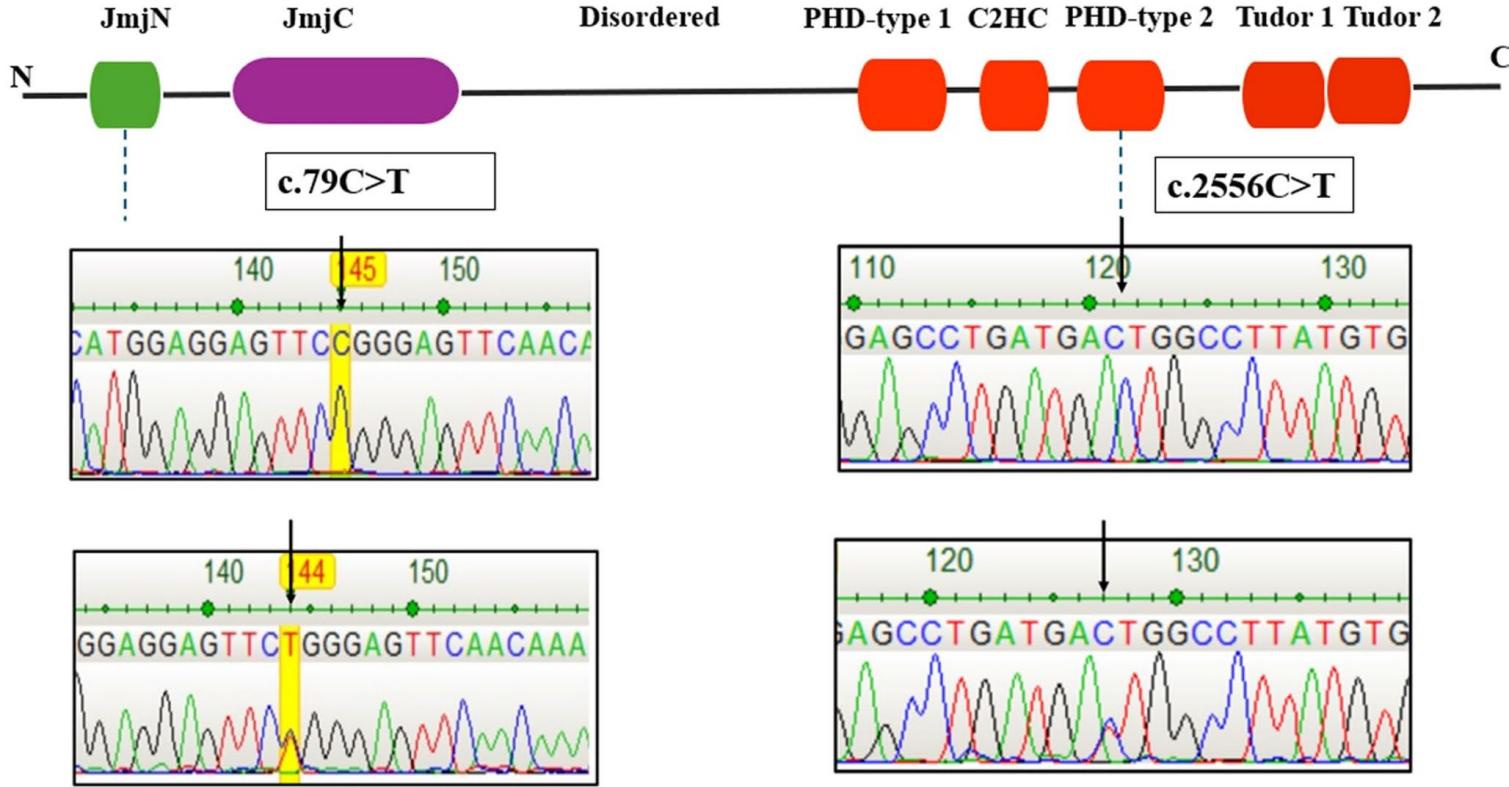
Structural representation of KDM4C protein and location of identified variants (c.79 C > T in JmjN domain and c.2556 C > T in PHD-type 2 domain)

Sanger sequencing identified a rare missense mutation in *KDM4C* (c.79 C > T; p.Arg27Trp, R27W) within the JmjN domain (Fig. 1).

This variant, which has not previously been reported in RA, was detected in a 65-year-old male patient with a 15-year disease history. The patient exhibited a seropositive phenotype (RF, anti-CCP, and ANA), elevated CRP (20 mg/dL) and ESR (53 mm/h) levels, prolonged morning stiffness, and resistance to DMARD, steroid, and anti-TNF therapy. The patient had no diabetes, hypertension, or family history of rheumatologic disease. The R27W variant was absent in controls and extremely rare globally (MAF < 0.01).

The p.Arg27Trp (R27W) variant lies within the JmjN region, which contributes to the structural context of the catalytic module in KDM4-family proteins. Given its rarity and absence in our control set, we considered it a candidate finding of interest. However, this observation is based on a single carrier, and computational predictions were not fully concordant across tools. Therefore, we report R27W descriptively and refrain from inferring disease causality or functional impairment without experimental validation.

### Synonymous variant in the PHD-type 2 domain

A synonymous substitution in *KDM4C* (c.2556 C > T) located in the PHD-type 2 domain was identified in patients with RA. Genotype frequencies were 33% CC, 54% CT, and 13% TT. The T allele frequency (40%) was higher than in controls (25%), but the difference was not statistically significant (*p* > 0.05). This variant (MAF = 0.43) is classified as a polymorphism in MutationTaster and has not previously been associated with RA. However, the homozygous presence of this polymorphism in global genomic variation databases, such as HapMap and the 1000 Genomes Project, suggests potential functional relevance. Therefore, its possible biological significance should not be overlooked. The PHD-type 2 domain acts as an epigenetic “reader” of histone modifications, facilitating chromatin targeting by *KDM4C*. Although synonymous, the c.2556 C > T variant may influence mRNA stability, codon usage, or translational efficiency. Through these mechanisms, it may indirectly affect protein function. Such seemingly neutral variants in reader domains may nonetheless have functional relevance, warranting further investigation in RA pathogenesis.

#### *In Silico* prediction of the R27W variant

**Fig. 2:**
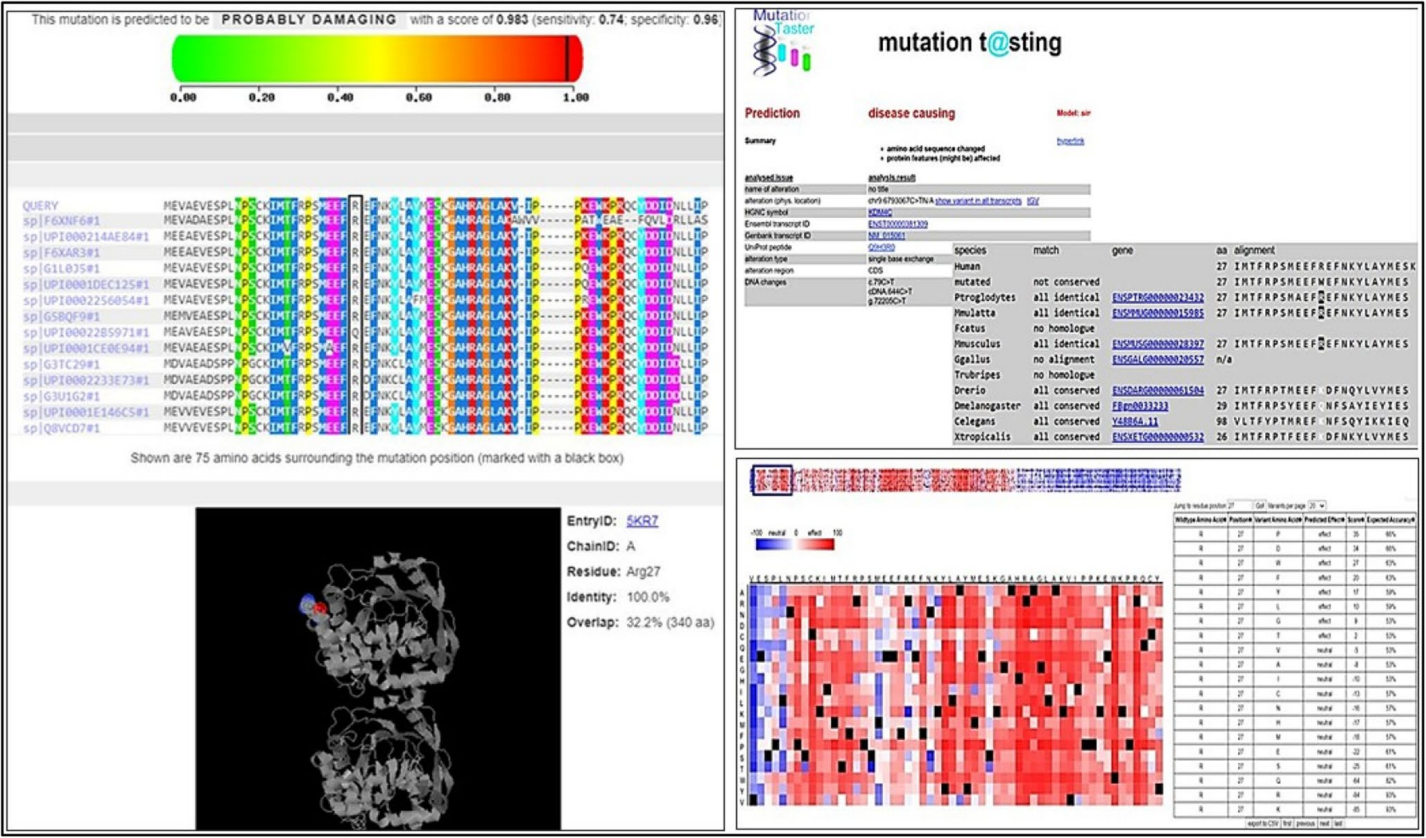
In silico prediction results for the R27W mutation using MutationTaster, SNAP2, and PolyPhen-2

The potential functional consequences of the R27W variant were extensively evaluated using multiple in silico prediction tools. MutationTaster classified the variant as “disease-causing”. PolyPhen-2 predicted it to be “probably damaging”, and the SNAP2 algorithm labeled it as having a functional “effect” (Fig. 2).

These concordant predictions collectively suggest that the variant may have potentially deleterious effects on the protein’s structure and function. Furthermore, this variant has not been previously reported in reference genetic databases, such as the Human Gene Mutation Database (HGMD). It has also not been associated with RA in the existing literature, underscoring its novelty and potential clinical relevance.

**Fig. 3:**
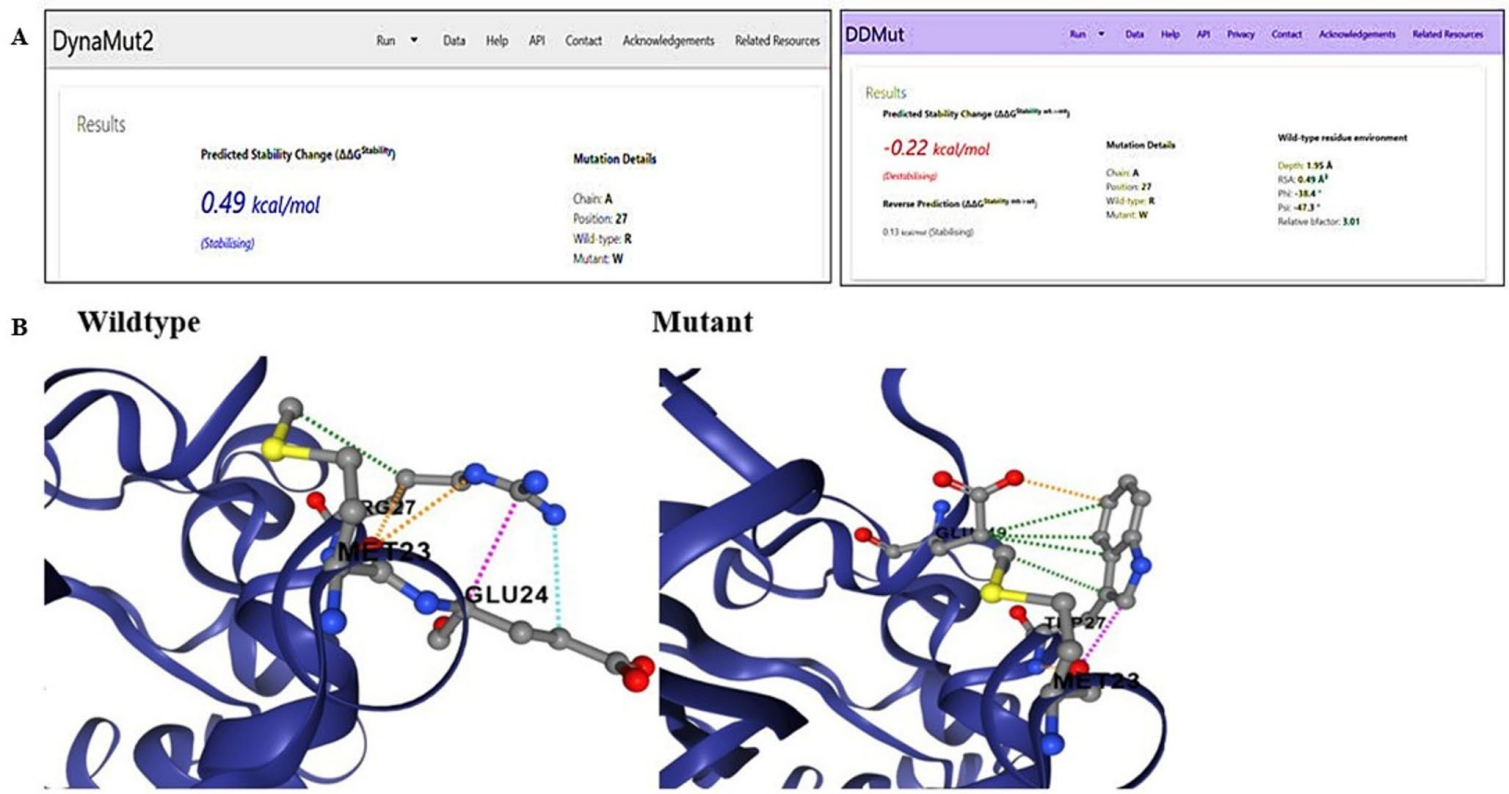
A Structural stability analysis of R27W mutation with DynaMut2 (ΔΔG = + 0.49 kcal/mol) and DDMut (ΔΔG = −0.22 kcal/mol). B dynamut prediction results for KDM4C

To investigate the structural consequences of the R27W missense variant located in the JmjN domain of the *KDM4C* gene, various computational tools were used to assess its effects on protein stability and pathogenicity. According to DynaMut2, the mutation was predicted to have a stabilizing effect on the protein; a ΔΔG value of + 0.49 kcal/mol (Fig. 3) suggests that the amino acid substitution may lead to an increase in local rigidity [44]. In contrast, the DDMut prediction model classified the mutation as mildly destabilizing, and a ΔΔG value of-0.22 kcal/mol suggests the possibility of local structural distortions or loss of dynamic flexibility [40].

These partially contradictory results suggest that the mutation may not significantly alter overall protein folding. However, it could still affect biological activity by subtly altering the conformation of functionally critical domains or intramolecular interactions.

According to MutPred2 analysis, the predicted probability of pathogenicity for the p.Arg27Trp variant is 0.197, which is below the generally accepted threshold of 0.5. This result suggests that the variant is unlikely to have a strong pathogenic effect based on current computational criteria. Therefore, the in silico findings should be interpreted cautiously and do not provide sufficient evidence to support a direct functional or causal role in rheumatoid arthritis in the absence of experimental validation.

It is noteworthy that this variant resides in the JmjN domain, which stabilizes the JmjC catalytic region through enzyme homodimerization. The substitution of arginine with tryptophan introduces a bulky, non-polar residue in place of a positively charged side chain. This change may disrupt enzyme homodimerization and impair histone demethylase activity without causing major structural instability. Although in silico analyses alone cannot confirm pathogenicity, several lines of evidence support the potential functional relevance of this variant. These include its rarity (MAF < 0.01), absence in the control group, localization within a critical functional domain, and association with reduced KDM4C expression in patients with RA. Thus, this variant represents a candidate of interest for further validation through structural modeling, protein–protein interaction assays, and cell-based functional studies.

### Expression analysis of ***KDM4C*** in RA patients

**Fig. 4:**
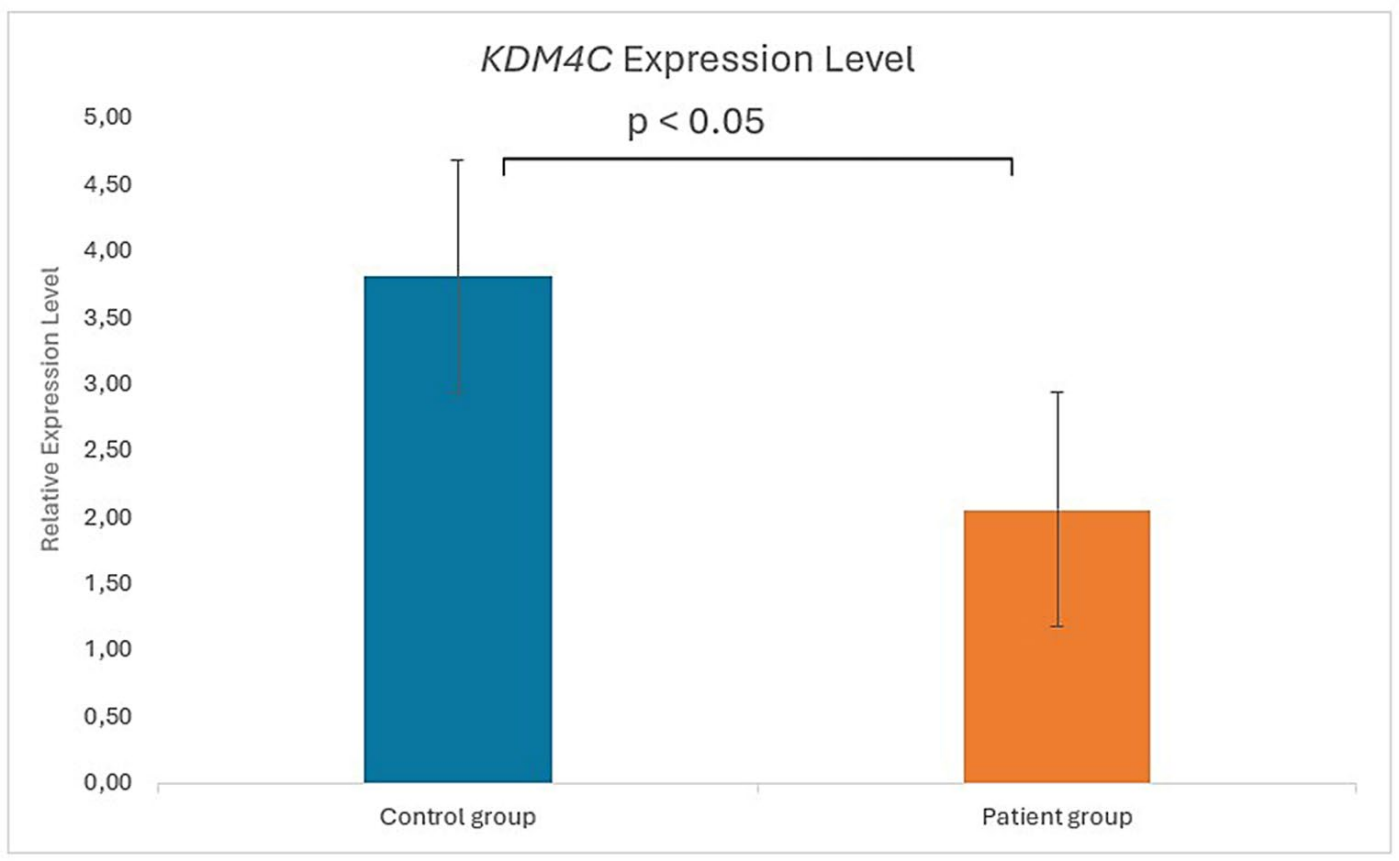
Relative *KDM4C* gene expression levels in RA patients and healthy controls normalized to GAPDH

RT-qPCR analysis of peripheral blood samples (30 RA patients, 10 controls) demonstrated significantly reduced *KDM4C* expression in RA, normalized to GAPDH (Fig. 4).

Reduced KDM4C transcript levels were observed in patients with rheumatoid arthritis compared with healthy controls. In parallel, a rare missense variant affecting the JmjN domain was identified in a single patient. While these findings suggest that both genetic variation and altered gene expression may be present in RA, the current data do not allow direct inference of functional impairment. Additionally, a synonymous c.2556 C > T variant was identified in the PHD-type 2 domain. Although silent, such substitutions may affect mRNA stability, codon usage, or translational efficiency. Given the PHD domain’s role as an epigenetic “reader” of histone modifications, even subtle alterations may indirectly perturb chromatin targeting and protein activity, warranting further investigation. Accordingly, the expression and variant data are reported descriptively, and no conclusions regarding enzymatic dysfunction or disease causality are drawn in the absence of experimental validation.

### Treatment profiles of RA patients

Among the 30 RA patients, five received DMARD monotherapy, most commonly methotrexate (MTX; *n* = 3), sulfasalazine (SSZ; *n* = 2), and leflunomide (*n* = 2). One patient (No. 17) was treated with steroid alone (Prednisolone DR 10 mg). Three patients received anti-TNF monotherapy-golimumab in two cases and etanercept in one. A single patient was treated with baricitinib, a JAK inhibitor.

Fourteen patients were managed with DMARD + steroid combinations, most frequently MTX + SZ+prednisone (*n* = 3) or leflunomide + SSZ+prednisone (*n* = 2). Other regimens included MTX+prednisone (*n* = 2), leflunomide+prednisone (*n* = 2), leflunomide+hydroxychloroquine+ prednisolone (*n* = 1), MTX+naproxen (*n* = 1), leflunomide+prednisolone (*n* = 1), and triple DMARD+prednisone (*n* = 2).

Among four patients receiving a DMARD+anti-TNF regimen without steroids, two were treated with MTX+golimumab and one with hydroxychloroquine+etanercept; the remaining patient received an individualized DMARD-biologic combination. Two patients underwent triple therapy (DMARD+steroid+anti-TNF), both including golimumab and prednisone, combined with leflunomide or MTX.

## Discussion

Rheumatoid arthritis is a chronic autoimmune disease characterized by persistent inflammation driven by genetic predisposition, environmental exposures, and immune dysregulation. Beyond genetic variation, epigenetic mechanisms have gained recognition as key contributors to disease onset and progression. The functional state of histone-modifying enzymes has emerged as a novel molecular target in RA and other immune-mediated disorders [10, 34].

*KDM4C*, a member of the histone demethylase family, is recognized as a central epigenetic regulator of chromatin remodeling and transcriptional programs in immune cells [5]. Consistent with previous evidence linking *KDM4C* to epigenetic regulation and disease susceptibility, the identification of a disease-segregating germline variant in a cancer-prone family highlights its potential pathogenic significance. These findings support the concept that rare *KDM4C* variants may contribute to disease risk by disrupting chromatin remodeling mechanisms (Ktainen et al. [18]).

In this study, structural mutations and reduced expression identified in the KDM4C gene were observed in association with rheumatoid arthritis, and the potential contribution of this gene to disease mechanisms was discussed. In the autoimmune disease samples examined, both structural abnormalities and a significant decrease in expression at the transcriptional level were observed in the *KDM4C* gene.

One of the identified mutations was a missense variant located in the JmjN domain, which is required for enzymatic activity. The second mutation was a synonymous variant located in the PHD-type 2 domain, which plays a role in chromatin targeting. A missense mutation in the JmjN domain disrupts the protein’s ability to form homodimers, leading to the loss of KDM4C’s demethylase activity [27]. This disruption leads to the accumulation of repressive histone marks, particularly H3K9me3. As a consequence, chromatin adopts a closed conformation, resulting in silencing of immune-related genes. Indeed, it has been previously shown that *KDM4C* plays a role in the control of B cell proliferation and immune response, and disruptions in this pathway contribute to autoimmunity [14]. However, no functional assays were performed in this study to directly assess H3K9me3 levels, *KDM4C* demethylase activity, protein stability, or homodimerization. Therefore, the proposed mechanistic interpretation is based on domain context and prior literature rather than direct experimental validation. Nevertheless, the tissue- and cell-type–specific nature of RA should be carefully considered. Follow-up studies in synovial tissue–derived cells and defined immune subsets will be required to determine whether *KDM4C* downregulation is driven by specific pathogenic cell populations.

The PHD-type 2 domain acts as an epigenetic “reader,” recognizing histone methylation marks and guiding *KDM4C* to chromatin targets [28]. Although synonymous variants do not change the amino acid sequence, they may influence mRNA folding, translation dynamics, or ribosomal pausing, thereby affecting protein conformation or efficiency [15]. However, for *KDM4C*, there is currently no direct experimental evidence demonstrating that synonymous substitutions within the PHD-type 2 domain affect chromatin recognition or binding fidelity. Therefore, any potential functional relevance of this variant should be considered speculative and hypothesis-generating.

Another important aspect of the findings is the significant decrease in *KDM4C* gene expression seen in patients. Reduced *KDM4C* expression leads to an imbalance in histone demethylases and, consequently, the accumulation of suppressive methylation marks. As a result, genes involved in immune regulation can be silenced, inflammatory responses can go uncontrolled, and autoimmune reactions can be triggered. Indeed, several studies have shown that *KDM4C* controls the activation and proliferation of immune cells through epigenetic regulation [14].

Literature indicates that reduced *KDM4C* expression is not solely driven by local genetic variation but also reflects broader epigenetic regulatory mechanisms. For instance, Kato et al. reported that rare copy number losses (CNVs) in KDM4C are significantly associated with schizophrenia and autism spectrum disorders. In patient-derived cell lines, these CNVs were accompanied by decreased *KDM4C* expression and altered histone methylation patterns. These findings suggest that structural variations in *KDM4C* can disrupt neuronal development through epigenetic mechanisms, contributing to neurodevelopmental pathology [19].

Studies in mouse models have shown that functional loss of the KDM4 family significantly reduces the proliferative and self-renewal capacity of hematopoietic stem cells [2]. This phenotypic change leads to the accumulation of genetic silencing mechanisms and repressive histone marks such as H3K9me3, resulting in reduced expression of vital genes in HSCs. These findings suggest that *KDM4C* plays a fundamental role not only in the epigenetic control of gene expression but also in maintaining tissue homeostasis.

Our findings indicate that both structural and regulatory variants in *KDM4C* may contribute to multilayered epigenetic dysregulation in RA. These alterations include a missense mutation in the JmjN domain, a synonymous variant in the PHD-type 2 domain, and reduced *KDM4C* gene expression. The JmjN substitution is predicted to impair homodimerization and potentially reduce demethylation of repressive H3K9me3, promoting transcriptional silencing. In parallel, the synonymous variant in the PHD-type 2 domain does not alter the protein sequence, and its functional relevance remains uncertain. Although synonymous variants in other genes have been reported to influence translation efficiency, mRNA stability, or ribosomal pausing, no *KDM4C*-specific experimental evidence currently supports an effect on chromatin targeting. Accordingly, any potential functional role of this variant should be regarded as speculative and hypothesis-generating [25, 42].

Collectively, these structural and functional impairments indicate a weakened epigenetic regulatory role of *KDM4C* in immune cells. This effect may be particularly relevant in cellular subpopulations such as synovial fibroblasts and B cells, which play pivotal roles in RA pathophysiology. The significant downregulation of *KDM4C* expression may result in insufficient suppression of pro-inflammatory genes, thereby contributing to the persistence of chronic inflammation within the synovial tissue microenvironment.

Accordingly, *KDM4C* emerges not only as a key molecule for elucidating the molecular mechanisms underlying RA but also as a promising candidate for diagnostic biomarker development and epigenetically targeted therapeutic strategies. Current treatment approaches in RA, particularly biologic DMARDs, have significantly improved disease control. However, refractory cases remain a challenge, highlighting the unmet need for new molecular targets [4]. In this context, epigenetic regulators such as *KDM4C* may represent complementary or alternative therapeutic avenues to address this gap.

A key limitation of this study is the small control cohort, which constrains accurate estimation of population-level frequencies, particularly for rare variants. This limitation also reduces the statistical power to detect subtle genetic associations. Future studies with larger and genetically diverse controls are required to validate these findings and refine their clinical significance. Moreover, functional analyses of *KDM4C* variants in advanced experimental models will be essential to delineate the epigenetic architecture of rheumatoid arthritis. Such studies, particularly in RA-relevant immune cell contexts, may inform the development of personalized therapeutic strategies.

### Limitations of the study

The most important limitation of the present study is the relatively small sample size, including 30 patients with rheumatoid arthritis and 10 healthy controls. No formal a priori power calculation was performed, as the primary aim of this study was exploratory and hypothesis-generating rather than definitive association testing. This limited cohort restricts statistical power and precludes robust estimation of effect sizes, particularly for rare genetic variants. Accordingly, the genetic findings of this study should be interpreted as exploratory and hypothesis-generating rather than conclusive.

The missense variant c.79 C > T (p.Arg27Trp; R27W), although absent in controls, extremely rare in population databases, and located within the functionally critical JmjN domain, was identified in a single RA patient. Therefore, no definitive conclusions can be drawn regarding its clinical significance, disease penetrance, or contribution to overall RA risk. Single-carrier observations are inherently insufficient for formal association testing and cannot establish causality in complex diseases. These limitations are consistent with well-recognized methodological challenges in rare-variant association studies of complex autoimmune diseases.

The pathogenic relevance of the R27W variant cannot be established based solely on in silico predictions, especially given the inconsistency among tools. Functional assays are essential to determine its effect on KDM4C enzymatic activity and protein function, and future studies should incorporate in vitro and/or cell-based experiments to evaluate the biological impact of this substitution. Although the p.Arg27Trp variant was evaluated using multiple in silico tools, no functional assays were performed. Therefore, no direct conclusions regarding pathogenicity or causal involvement in rheumatoid arthritis can be drawn. Functional validation (e.g., H3K9me3 quantification, demethylase activity and interaction assays) is required to test the proposed mechanistic hypotheses.

Rare variant analysis in polygenic and multifactorial disorders such as RA typically requires either very large sequencing-based case-control cohorts employing gene-based burden or kernel tests, or family-based study designs to demonstrate segregation with disease. While family-based segregation analysis can provide strong support for pathogenicity, its application in RA is frequently limited by genetic heterogeneity, reduced penetrance, and the lack of large multiplex families. These constraints were present in the current study and prevented evaluation of variant segregation.

Furthermore, whole blood represents a heterogeneous cellular compartment, and no peripheral blood mononuclear cell subset enrichment, cell sorting, or deconvolution approaches were applied in this study. Therefore, the observed *KDM4C* expression differences should be interpreted as aggregate systemic signals and cannot be attributed to specific disease-relevant cell populations such as synovial fibroblasts or defined B-cell subsets. In addition, the functional relevance of the synonymous variant identified in the PHD-type 2 domain remains uncertain. Although synonymous variants in other genes have been reported to influence mRNA structure, translation dynamics, or protein function, there is currently no *KDM4C*-specific experimental evidence demonstrating that codon usage or mRNA folding within the PHD-type 2 domain is rate-limiting or directly affects chromatin targeting. Therefore, any potential functional implication of this synonymous variant should be regarded as speculative and hypothesis-generating, and requires experimental validation.

In addition, *KDM4C* expression was measured in peripheral blood of patients undergoing heterogeneous but ongoing antirheumatic therapy, and with the limited sample size we were not able to statistically adjust for the effects of specific DMARDs, glucocorticoids or biologic agents; therefore, we cannot fully disentangle disease-intrinsic epigenetic alterations from treatment-related modulation of gene expression.

Future studies should therefore aim to validate these findings in larger, multi-center cohorts and, where possible, incorporate family-based analyses and functional assays to assess the biological consequences of candidate *KDM4C* variants. Despite these limitations, the present study provides initial, hypothesis-generating clinical and molecular observations that support a potential role of *KDM4C* dysregulation in rheumatoid arthritis pathogenesis and establish a rationale for further investigation in larger and more comprehensive study designs.

## Data Availability

The datasets generated during the current study and/or analyzed are available from the authors upon reasonable request.
